# Degradation of reactive red (B-3BF) dye wastewater using UV irradiation (254/185 nm) with sodium persulfate in a pilot UV device

**DOI:** 10.1038/s41598-024-62967-3

**Published:** 2024-05-27

**Authors:** Chao Wang, Yongqiang Li, Junmin Wan, Yi Hu, Yi Huang, Jiangen Qiu

**Affiliations:** 1 College of Textile Science and Engineering (International Institute of Silk), Zhejiang Sci-Tech University, No. 928, 2nd Street, Xiasha Higher Education Zone, Hangzhou City, 310018 Zhejiang Province China; 2 Tongxiang Research Institute, Zhejiang Sci-Tech University, No. 1488, Development Avenue, Tongxiang City, Jiaxing City, 314599 Zhejiang Province China; 3 School of Materials Science and Engineering, Zhejiang Sci-Tech University, No. 928, 2nd Street, Xiasha Higher Education Zone, Hangzhou City, 310018 Zhejiang Province China

**Keywords:** Pilot UV, UV irradiation, Sodium persulfate, Degradation percentage, Energy consumption, Dye wastewater, Environmental sciences, Chemistry, Engineering

## Abstract

Two low-pressure ultraviolet (UV) lamps at 185/254 nm with sodium persulfate in a pilot UV device were utilized for the degradation of reactive red (B-3BF) dye wastewater compared with two UV lamps at 185/185 nm and two UV lamps at 254/254 nm. The degradation performances of UV irradiation (254/185 nm) with sodium persulfate under different degradation times, flow rates, initial pH, initial Na_2_S_2_O_8_ concentrations and initial dye concentrations were investigated. The experimental results illustrated that the degradation percentage of B-3BF dye could reduce to 90.42% with the energy consumption of 85.1 kWh/kg and the residual dye concentration of 1.92 mg/L by UV irradiation (254/185 nm) with initial Na_2_S_2_O_8_ concentration of 1.5 mmol/L and initial dye concentration of 20 mg/L. In addition, degradation performance of B-3BF dye wastewater by UV irradiation (254/185 nm) with sodium persulfate was more effective than those of UV irradiation (254/254 nm) and UV irradiation (185/185 nm). Therefore UV irradiation (254/185 nm) with sodium persulfate was promising for the degradation of B-3BF dye wastewater.

## Introduction

Reactive red dye is widely used in the textile industry. A large amount of textile wastewater containing dyestuffs can be generated during the textile manufacturing processes, which can be harmful to human health and ecosystems^1^. Physical and chemical treatment methods followed by biological technology are the most widely used in the textile wastewater, such as chemical setting, adsorption, membrane filtration. Generally, the dye can not be effectively destroyed by conventional treatment methods, which may generate harmful or toxic substances^2^. Advanced oxidation processes have been studied extensively in chemically degrading various organic contaminants, such as electrochemical oxidation, photocatalytic oxidation^3,4^. Hydrogen generated in electrochemical oxidation is still a safe issue, which requires further safety protection measures or treatment methods. Most of photocatalytic oxidations required the introduction of photocatalysts, which increase the cost of the wastewater treatment^5–7^.

Ultraviolet (UV) irradiation is based on hydroxyl radicals or persulfate radicals utilizing UV lamps combined with O_3_, H_2_O_2_, chlorine or persulfate for the degradation of organic pollutants^8,9^. UV lamps at 185 nm and 254 nm are the main lamps used in UV irradiation for the wastewater treatment^10,11^. Studies have reported the effective degradation of some organic contaminants could be achieved by UV at 254 nm or UV at 185 nm with oxidizing agents^12^. The degradation of Brilliant Blue FCF dye by UV/chlorine at 254 nm was carried out to achieve the elimination rate of over 95% during 30 min reaction time at chlorine dosage of 0.6 mmol/L, and the scavenging assay showed that chlorine radicals of 17% and dichlorine radicals 10.4% had minor role in degradation of the dye compared with the hydroxyl radicals contribution of 66.9%^13^. The degradation removal of reactive orange 122 (RO122) azo dye was reported to be 95% by (UV/ H_2_O_2_) at 100 mg/L H_2_O_2_ for 60 min^14^. Researches have revealed that UV radiation at 185 nm was capable of degrading some organics by itself due to its energy^15,16^. In addition, ozone can be generated by O_2_ absorbed in UV radiation at 185 nm.

Persulfate combined with UV has been proved to be a powerful oxidizing agent for the degradation of some organic contaminants due to high yield of sulfate radicals formation and its longer half-life compared to other reactive oxygen species^17^. The conversion of Acid Blue 129 could reach 87% at peroxydisulfate concentration of 2.5 mmol/L after 5 min and the research results illustrated that UV-activated peroxymonosulfate is more efficient for dye degradation than UV-activated peroxydisulfate^18^. UV activation could reduce the formation of by-products and hence could be more environmentally friendly. It was reported that degradation percent of Rhodamine B by UV/persulfate could reach 87% in 15 min^19^. The pilot-scale UV/O_3_ pressurization process was performed for actual high-salt textile wastewater of reverse osmosis and achieved the decolorization of 85%, removal percentage of 43.2% for chemical oxygen demand (COD) at an O_3_ dosage of 200 g/t and a pressure of 0.2 MPa^20^. However, the addition of O_3_ required the ozone generator, which increased the cost of UV/O_3_.

So far, UV irradiation at 185/254 nm has obtained attention in the degradation of organic pollutants, such as polyfluoroalkyl substances^21^. Most of investigations of UV irradiation for wastewater treatment were carried out in a laboratory scale. In present study, pilot experiments were performed for the wastewater containing dye, which could be more valuable for the industrial application.

This study was undertaken to compare the degradation performances of UV irradiation at 185 nm, UV irradiation at 254 nm, UV irradiation at 185/254 nm for reactive red (B-3BF) dye wastewater to verify the assumption that the UV irradiation at 185/254 nm has better degradation rate of B-3BF dye than that of UV irradiation at 185 nm or 254 nm. The degradation parameters of the UV irradiation at 185/254 nm were optimized under different degradation times, flow rates, initial pH, initial Na_2_S_2_O_8_ concentrations and initial dye concentrations to investigate the degradation performance of the pilot UV device.

## Experimental

### Materials and chemicals

**Figure 1:**
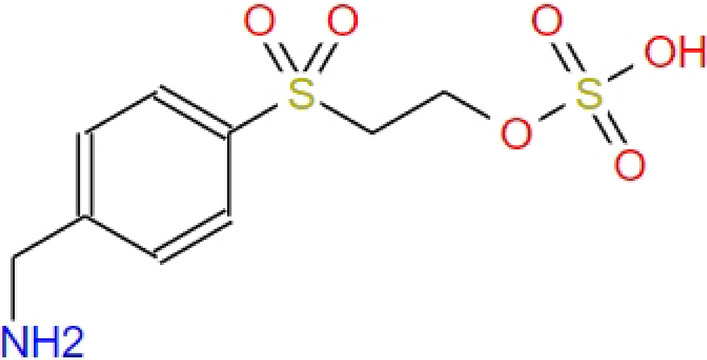
Chemical structure of B-3BF dye.

The commercial low-pressure UV lamps of 80W at 185 nm and 254 nm were purchased from Suzhou Hemingway Environmental Protection Equipment Co., Ltd. The low-pressure UV lamps were stalled in a pilot UV device manufactured by Guangzhou Anders Electromechanical Equipment Co., Ltd. Reactive red (B-3BF) dye was purchased from Jize County Shunde Dye Distribution Co., Ltd. Sulfuric acid (H_2_SO_4_), sodium hydroxide (NaOH) and sodium persulfate (Na_2_S_2_O_8_) were of analytical grade and purchased from Weisi (Beijing) Experimental Supplies Co., Ltd. The dye wastewater was prepared by the reactive red (B-3BF) dye and deionized water. The chemical structure of B-3BF dye is given in Fig. 1

### Experimental apparatus

**Figure 2:**
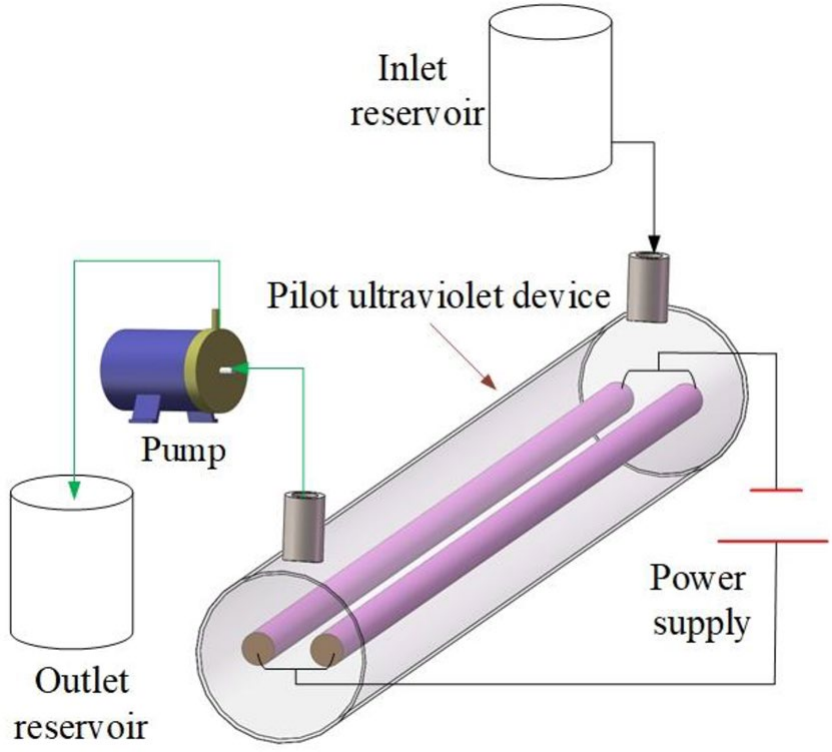
Diagram of the experimental apparatus.

As shown in Fig. 2, the diagram of the experimental apparatus for the degradation of the dye wastewater consists of a magnetic pump (MP-20RM) from Shanghai Xinxishan Bengye Co., Ltd, a pilot UV device with an ultraviolet lamp at 185 nm and an ultraviolet lamp at 254 nm to perform UV irradiation (185/254 nm). When experiments with UV irradiation (185/185 nm), two ultraviolet lamps at 185 nm were installed in the UV device. As for experiments with UV irradiation (254/254 nm), there were two UV lamps in 254 nm in the UV device.

### Experimental design

The B-3BF dye wastewater of 25 L flowed from the inlet reservoir to the pilot UV device and then flowed into the outlet reservoir by the pump during one time experiment. The dye wastewater was repeatedly flowed from the inlet reservoir to the pilot UV device to be degraded for the next degradation time. The temperature of dye wastewater was ranged from 30 to 35 °C when degradation time, dye wastewater flow rate, initial pH, initial Na_2_S_2_O_8_ concentration and initial dye concentration were optimized. The initial UV degradation was operated at flow rate of 500 L/h, initial pH of 7.8, initial Na_2_S_2_O_8_ concentration of 1.5 mmol/L and initial dye concentration of 20 mg/L. The volume of the pilot UV device was 7 L with the corresponding average retention time of 0.84 min for one degradation time when the dye wastewater flowed through the pilot UV device at flow rate of 500 L/h. The pH of the B-3BF dye wastewater was adjusted by 0.1 mol/L NaOH solution and 0.1 mol/L H_2_SO_4_ solution. The wastewater samples for analysis were extracted at the outlet reservoir when each experiment was finished.

### Analytical methods

The UV visible multi-parameter water quality tester (LH-3BA, China) was used to analyze the dye’s absorbance at its λmax = 545 nm and the dye concentration was obtained according to the relation between dye’s absorbance and dye concentration.

Degradation percentage of the B-3BF dye (C_d_, %) is calculated using Eq. (1):

1 $${\text{C}}_{{\text{d}}} = \frac{{{\text{c}}_{{\text{o}}} - {\text{c}}_{{\text{t}}} }}{{{\text{c}}_{{\text{o}}} }} \times 100\%$$

where c_t_ and c_o_ are the concentration of the B-3BF dye after degradation and before degradation, respectively, mg/L.

Since the energy was consumed by the pilot UV device and the magnetic pump, the energy consumption per kilogram of the B-3BF dye (W_e_, kWh/kg) is calculated using Eq. (2):

2 $${\text{W}}_{{\text{e}}} = \frac{{\left( {2 \times {\text{P}}_{{\text{u}}} + {\text{P}}_{{\text{m}}} } \right){\text{t}}/3600/1000}}{{\left( {{\text{vt}}/60} \right) \times ({\text{c}}_{{\text{o}}} - {\text{c}}_{{\text{t}}} )/1000/1000}} = \frac{{50 \times \left( {2 \times {\text{P}}_{{\text{u}}} + {\text{P}}_{{\text{m}}} } \right)}}{{3 \times {\text{v}} \times ({\text{c}}_{{\text{o}}} - {\text{c}}_{{\text{t}}} )}}$$

where t is the total time when the UV irradiation is applied, s; v is the dye wastewater flow rate, L/h; P_u_ is the power of one UV lamp, 80 W; P_m_ is the power of the magnetic pump, 15 W.

## Results and discussion

### Comparison of different UV lamps on degradation performance

**Figure 3:**
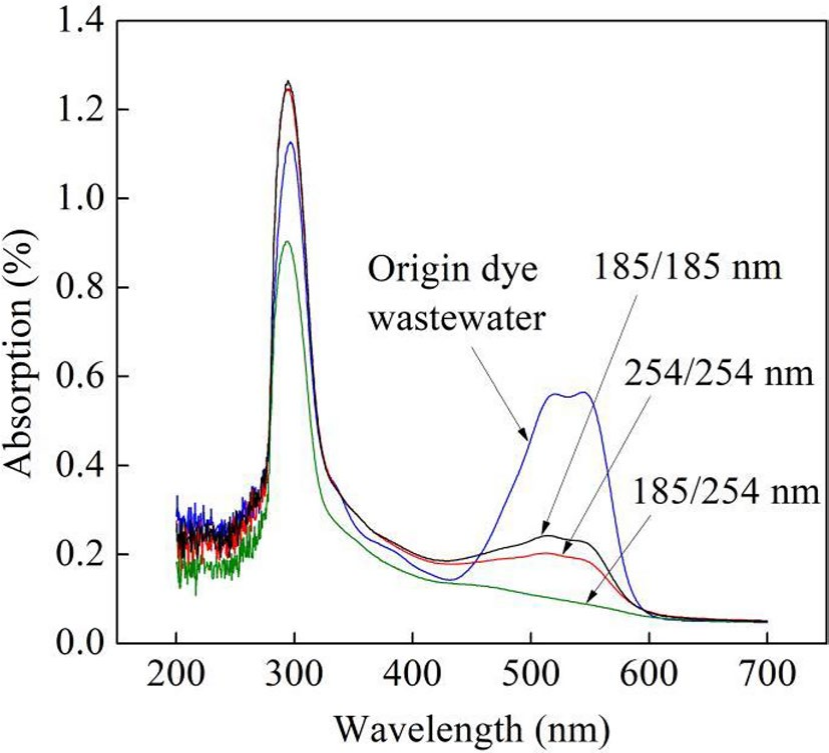
UV–visible spectrums of the dye wastewater by different UV irradiations with Na_2_S_2_O_8_.

**Figure 4:**
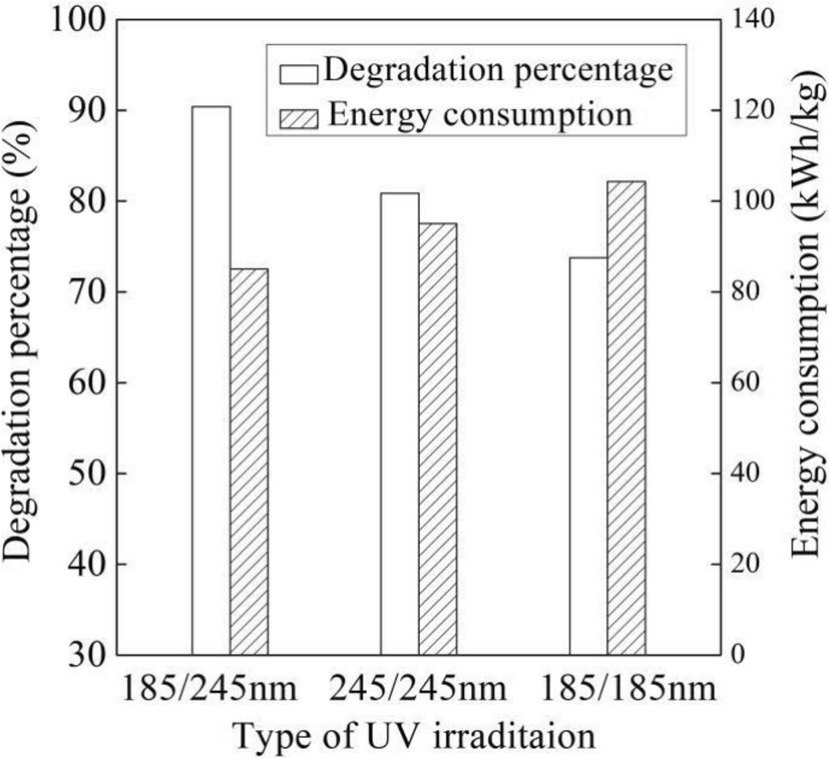
Degradation performance of different UV irradiations with Na_2_S_2_O_8_.

Figure 3 displays the UV–visible spectrums of the B-3BF dye wastewater by different UV irradiations with initial Na_2_S_2_O_8_ concentration of 15.0 mmol/L at six degradation times, flow rate of 500 L/h, initial pH of 7.8 and initial dye concentration of 20 mg/L. It can be observed from Fig. 3 that the dye wastewater contains the benzene ring at 296 nm, -SO_3_ group at 521 nm and the chromophore in the dye molecule at 543 nm^22,23^. The dye’s characteristic wavelength, 545 nm, experienced a rapid decrease due to the UV irradiation. Compared with UV irradiations by two UV lamps at 185 nm and two UV lamps at 254 nm, the peaks at 296 nm, 521 nm and 543 nm were further reduced by UV irradiation (185/254 nm) as shown in Fig. 3, which illustrated that the UV irradiation (185/254 nm) with sodium persulfate could have more effective degradation performance of B-3BF dye wastewater. The effective degradation performance of UV irradiation (185/254 nm) was demonstrated by analyzing the degradation percentage and the energy consumption of different UV irradiations, as reported in Fig. 4. The degradation percentage of UV irradiation (185/254 nm), UV irradiation (254/254 nm) and UV irradiation (185/185 nm) with sodium persulfate were 90.42%, 80.86% and 73.78% with the corresponding energy consumption of 85.1 kWh/kg, 95.1 kWh/kg and 104.3 kWh/kg, respectively.

Persulfate ion could be activated to form sulfate radical (${\text{SO}}_{4}^{{ - {\mathbf{ \cdot }}}}$) under UV irradiation (185 nm) and UV irradiation (254 nm) that was powerful oxidizing agents with its redox potential of 2.5 to 3.1 V^24^. In addition, the ozone could be generated in gas or water through direct photolysis of UV irradiation (185 nm). It can be seen from Figs. 3 and 4 that the degradation of B-3BF dye wastewater by UV irradiation (254/254 nm) was more effective than that by UV irradiation (185/185 nm), which illustrated that the degradation of B-3BF dye wastewater was mainly attributed to the sulfate radical. Additionally, it can be concluded that the activation of persulfate ion under UV irradiation (254 nm) was more effective than that under UV irradiation (185 nm). Besides, restricted by oxygen gas or dissolved gas in the dye wastewater, the generation of ozone by UV irradiation (185 nm) is limited to some extent. Compared with UV irradiation (254/254 nm), UV irradiation (185/254 nm) exhibited more effective degradation performances as shown in Figs. 3 and 4, which demonstrated that the UV irradiation (185/254 nm) combining the sulfate radical formed under UV irradiation and the generation of ozone under UV irradiation (185 nm) could achieve more effective degradation of the B-3BF dye wastewater. Additionally, although the oxygen content in the dye wastewater is limited, the introduction of ozone generated under one ultraviolet lamp at 185 nm improved the degradation performances compared with UV irradiation (185/185 nm) and UV irradiation (254/254 nm), which highlighted the benefit of UV irradiation (185/254 nm) for the degradation of B-3BF dye wastewater. As shown in Fig. 4, compared with UV irradiations (254/254 nm) and (185/185 nm), the degradation percentage of UV irradiation (185/254 nm) with sodium persulfate was higher with the lower energy consumption that was selected for the following experiments.

### The effect of degradation time and flow rate on degradation performance

**Figure 5:**
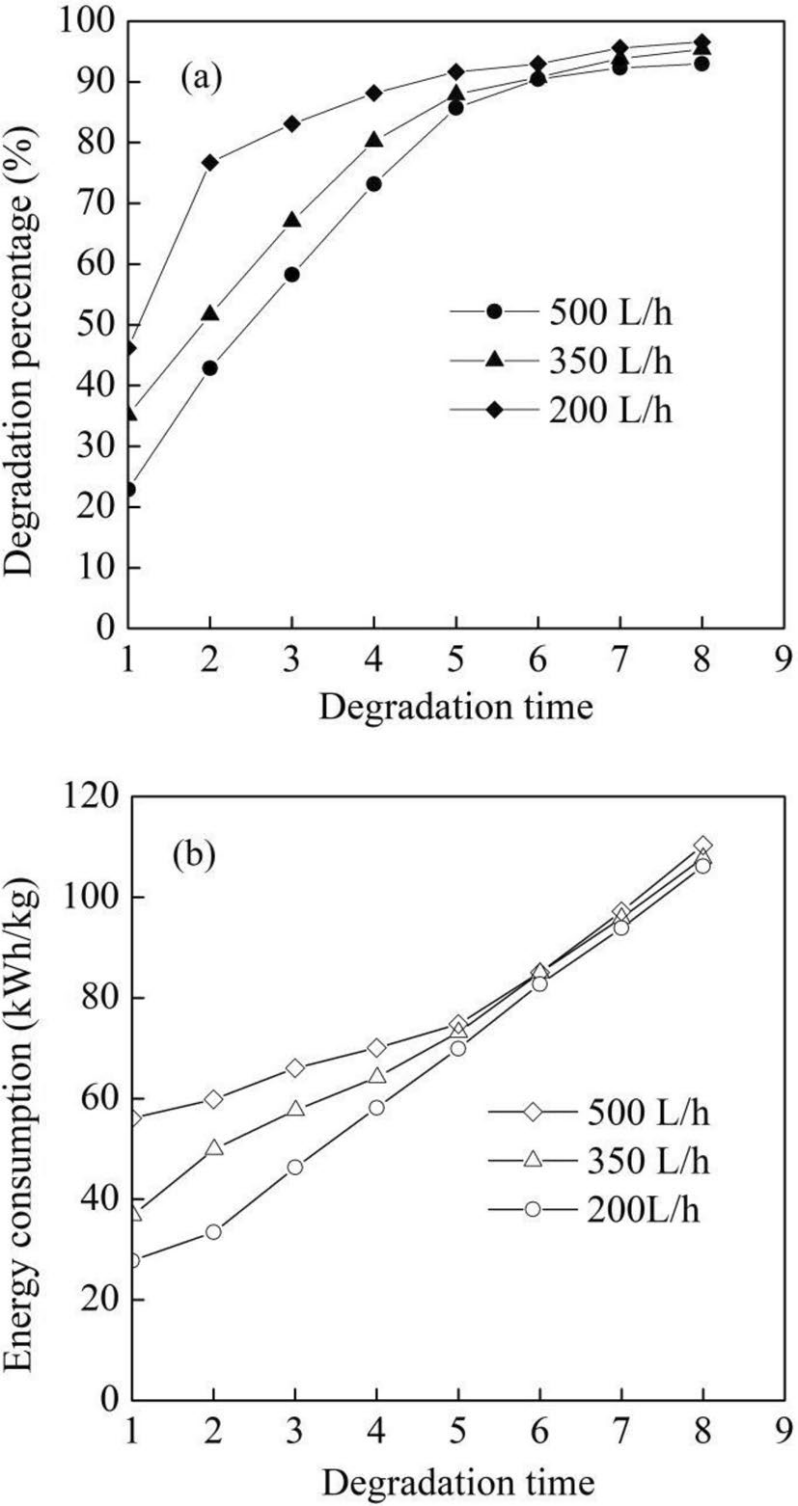
Degradation performance of different degradation times and flow rate. (**a**) Degradation percentage and (**b**) energy consumption.

As shown in Fig. 5a, the degradation percentage of the dye wastewater increased fast at first and then remained relatively stable under UV irradiation (185/254 nm) with the degradation time from one time to eight times at initial pH of 7.8, initial Na_2_S_2_O_8_ concentration of 15.0 mmol/L and initial dye concentration of 10 mg/L. Sulfate radical (${\text{SO}}_{4}^{{ - {\mathbf{ \cdot }}}}$) was generated under the UV lamp at 254 nm as shown in Eq. (3). On one hand, the degradation amount of the dye was limited by the amount of oxidants generated. On the other hand, the increase of degradation time meant the increase of contract time between the dye wastewater and the pilot UV device. When the degradation time increased from one time to eight times, the average contact time increased from 0.84 to 6.72 min at flow rate of 500 L/h, which caused that the more by-products were generated and inhibited the increase rate of degradation percentage. Therefore, the degradation percentage remained relatively stable at degradation time of six and reached 90.42%, 90.77% and 92.96% at the flow rate of 500 L/h, 350 L/h and 200 L/h, respectively shown in Fig. 5a.

3 $${\text{S}}_{2} {\text{O}}_{8}^{2 - } + hv \to {\text{SO}}_{4}^{{ - {\mathbf{ \cdot }}}}$$

Additionally, there existed more time for the mass transfer at the lower flow rate in the pilot UV device, which caused a larger amount of the dye was degraded. Therefore, the degradation percentage decreased with the flow rate and reached 92.67%, 95.38% and 96.57% at the flow rate of 500 L/h, 350 L/h and 200 L/h, respectively at eight degradation times shown in Fig. 5a. When the degradation time reached six, the degradation reaction of the dye was almost sufficient. Consequently, the difference of degradation percentage among the flow rates was small shown in Fig. 5a. It can be seen from Fig. 5b that the energy consumption gradually increased with the degradation time and reached 110.3 kWh/kg, 106.2 kWh/kg and 107.5 kWh/kg at flow rate of 500 L/h, 350 L/h and 200 L/h, respectively at eight degradation times. In addition, the energy consumption increased faster when the degradation time from six to eight due to the small amount of the degraded dye according to Eq. (2). The energy consumption at six degradation times was 85.1 kWh/kg, 84.7 kWh/kg and 82.7 kWh/kg at flow rate of 500 L/h, 350 L/h and 200 L/h, respectively. Obviously, there was not much difference of degradation percentage and energy consumption among the three flow rates at six degradation times.

The degradation efficiency of acid red 73 dye (AR73) reached 98.98% by the UV/hydrogen peroxide/peroxydisulfate system for 30 min in a 500 mL beaker under UV irradiation (254 nm)^25^. The contact time between the AR73 dye wastewater and the UV irradiation was 30 min with the dye wastewater confined in the beaker. However, it is widely used that textile wastewater continuously flows through the different treatment devices in industrial application^26^. Therefore the pilot UV device in this work can be introduced into the wastewater treatment process by the dye wastewater pipe connected with the UV device. Compared with the dye wastewater confined in the beaker, the pilot scale experiments with the dye wastewater flowing through the UV device shown in Fig. 2 could reflect the degradation performances of the dye wastewater in industrial application. The flow rate of dye wastewater was usually high in the industrial application. Although the degradation percentage at six degradation times was a little lower than that at eight degradation times, the energy consumption at six degradation times was much lower than that at eight degradation times. Considering the energy consumption in the industrial application, the six degradation times and the flow rate of 500 L/h were selected in the following experiments.

### The effect of initial pH on degradation performance

**Figure 6:**
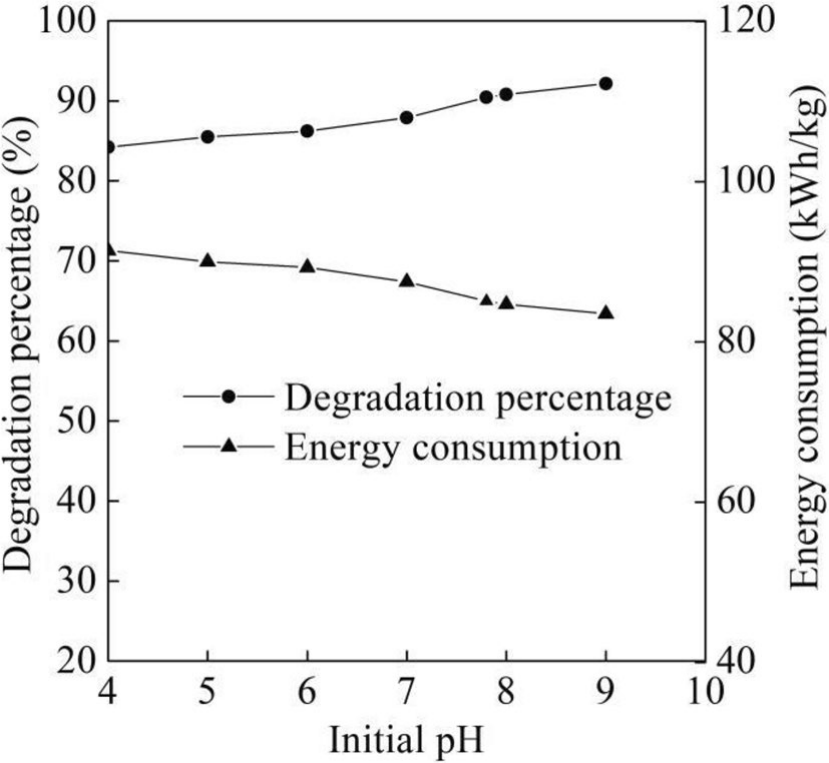
Degradation performance of different initial pH.

The alkaline activated persulfate systems have been used in the decomposition of organic compounds such as alkene, aromatic and carbon tetrachloride^27^. As shown in Fig. 6, the degradation percentage of the B-3BF dye wastewater gradually increased at pH from 4.0 to 7.0 and then increased a little fast from 87.9 to 92.13% at pH from 7.0 to 9.0 with six degradation times, flow rate of 500 L/h, initial Na_2_S_2_O_8_ concentration of 15.0 mmol/L and initial dye concentration of 20 mg/L. Persulfate could be activated by the alkaline^28^, which resulted in the rise of degradation percentage at pH from 4.0 to 9.0. It could be seen from Fig. 6 that the energy consumption gradually reduced from 91.4 to 83.5 kWh/kg at pH from 4.0 to 9.0. The change trend of degradation percentage with pH from 4.0 to 9.0 shown in Fig. 6 was consistent with the increase trend of removal percentage in COD and NH_3_-N of nanofiltration concentrated leachate by heat/UV (254 nm) activated persulfate^29^. On the contrary, sulfate radical was reported to be decomposed with the generation of hydroxyl radical^30^, as shown in Eq. (4).

4 $${\text{SO}}_{4}^{{ - {\mathbf{ \cdot }}}} + {\text{OH}}^{ - } \to {\text{SO}}_{4}^{2 - } + {\text{OH}} \cdot$$

Based on Eq. (4), with further increase in pH, the increased OH^−^ in wastewater tends to react with sulfate radical and generate hydroxyl radical. Based on the increase trend of the B-3BF dye wastewater at pH from 4.0 to 9.0 shown in Fig. 6, it could be proposed that OH· was predominant radical species with respect to B-3BF dye in the alkaline condition, which was also consistent with degradation performance of other organic contaminant by UV-activated persulfate oxidation^31^. Although the alkaline can activate the persulfate, the addition of alkaline increased the cost of wastewater treatment. In addition, the degradation percentage of the B-3BF dye wastewater at initial pH of 7.8 was 90.42% that was a little lower than that at initial pH of 9.0. Therefore, the initial pH of 7.8 was selected for the following experiments.

### The effect of initial Na_2_S_2_O_8_ concentration on degradation performance

**Figure 7:**
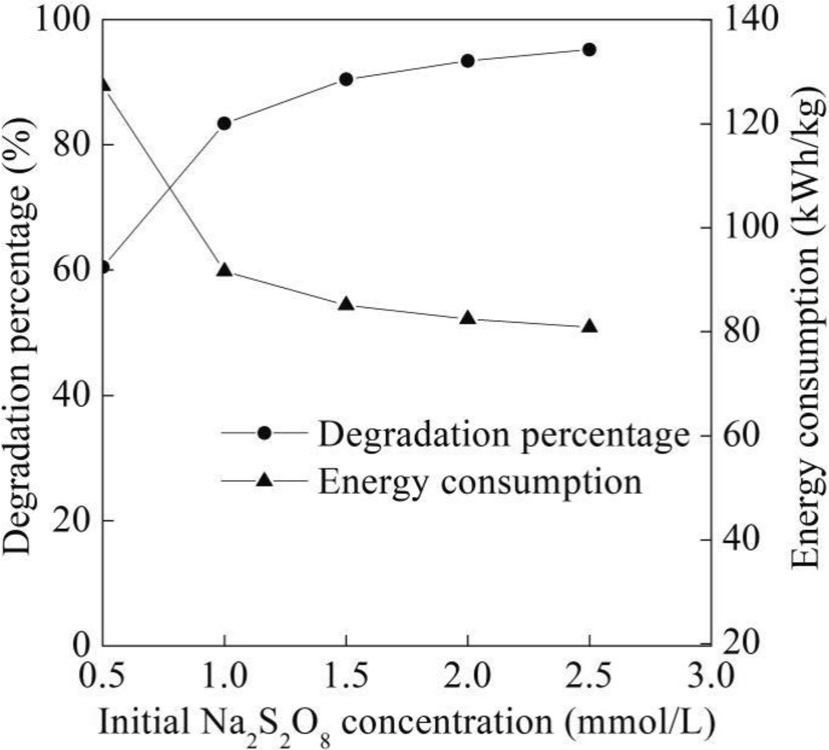
Degradation performance of different initial Na_2_S_2_O_8_ concentration.

It can be seen from Fig. 7 that the degradation percentage of the B-3BF dye wastewater increased fast at first with initial Na_2_S_2_O_8_ concentration from 0.5 to 1.5 mmol/L and then increased slowly with initial Na_2_S_2_O_8_ concentration from 1.5 to 2.5 mmol/L at six degradation times, flow rate of 500 L/h, initial pH of 7.8 and initial dye concentration of 20 mg/L. Generally, degradation percentage would be proportional to the amounts of sulfate radicals at low oxidant concentrations. In addition, the utilization of more oxidants may affect degradation percentage due to the self-scavenging effect^32^, as shown in Eq. (5). The chemical scavenging reactions between sulfate radical (${\text{SO}}_{4}^{{ - {\mathbf{ \cdot }}}}$) with sulfate radical (${\text{SO}}_{4}^{{ - {\mathbf{ \cdot }}}}$) are favored in the presence of sulfate radical (${\text{SO}}_{4}^{{ - {\mathbf{ \cdot }}}}$).

5 $${\text{SO}}_{4}^{{ - {\mathbf{ \cdot }}}} + {\text{SO}}_{4}^{{ - {\mathbf{ \cdot }}}} \to {\text{S}}_{2} {\text{O}}_{8}^{2 - }$$

As the initial Na_2_S_2_O_8_ concentration increased, the increase of sulfate radical concentration caused the gradual increase of degradation percentage of the B-3BF dye as shown in Fig. 7. When the degradation percentage of the B-3BF dye was beyond 90.42% at initial Na_2_S_2_O_8_ concentration of 1.5 mmol/L, the self-scavenging effect of Na_2_S_2_O_8_ may inhibited the increase rate of degradation percentage. The self-scavenging effect of Na_2_S_2_O_8_ was reported in decolorization of Reactive Red 239 by UV-C activated peroxydisulfate^33^. There was not observable increase in decolorization efficiency of Reactive Red 239 with Na_2_S_2_O_8_ concentration beyond 2 mmol/L, which was consistent with Eq. (5). Additionally, as for degradation percentage beyond 90.42%, more intermediate products generated may lead to the drop of mass transfer between B-3BF dye and sulfate radical, which lowered the increase rate of the corresponding degradation percentage. Figure 7 illustrated that the energy consumption gradually decreased at first and then decreased slightly with the increase of initial Na_2_S_2_O_8_ concentration at six degradation times, flow rate of 500 L/h, initial pH of 7.8 and initial dye concentration of 20 mg/L. With the increase of initial Na_2_S_2_O_8_ concentration from 0.5 to 1.5 mmol/L, the gradual increase of degradation percentage lowered the energy consumption according Eq. (2). As the initial Na_2_S_2_O_8_ concentration increased over 1.5 mmol/L, the slight increase of degradation percentage illustrated the degraded B-3BF dye obtained a slight rise, which resulted in the low increase rate of the energy consumption.

The Na_2_S_2_O_8_ cost was about 5000 RMB per ton and the average cost of industrial electricity was 0.725 RMB/kWh as for the energy consumption in China. As for B-3BF dye per kilogram, operational cost (Mc, RMB/kg) including Na_2_S_2_O_8_ cost and industrial electricity cost is calculated using Eq. (6):

6 $$\begin{aligned} {\text{M}}_{{\text{c}}} & = {\text{W}}_{{\text{e}}} \times {\text{f}}_{{\text{e}}} + \frac{{\left( {{\text{vt}}/60} \right) \times {\text{C}}_{{{\text{Na}}_{2} {\text{S}}_{2} {\text{O}}_{8} }} \times {\text{M}}/10^{6} \times {\text{f}}_{{{\text{Na}}_{2} {\text{S}}_{2} {\text{O}}_{8} }} }}{{\left( {{\text{vt}}/60} \right) \times ({\text{c}}_{{\text{o}}} - {\text{c}}_{{\text{t}}} )/10^{6} }} \\ & = {\text{W}}_{{\text{e}}} \times {\text{f}}_{{\text{e}}} + \frac{{{\text{C}}_{{{\text{Na}}_{2} {\text{S}}_{2} {\text{O}}_{8} }} \times {\text{M}} \times {\text{f}}_{{{\text{Na}}_{2} {\text{S}}_{2} {\text{O}}_{8} }} }}{{({\text{c}}_{{\text{o}}} - {\text{c}}_{{\text{t}}} )}} \\ \end{aligned}$$

where t is the total time when the UV irradiation is applied, s; v is the dye wastewater flow rate, L/h; W_e_ is the energy consumption per kilogram of the B-3BF dye, kWh/kg; f_e_ is the average cost of industrial electricity, 0.725 RMB/kWh; C_Na2S2O8_ is the initial Na_2_S_2_O_8_ concentration, mmol/L; M is the molar mass of Na_2_S_2_O_8_, 238.105 g/mol; f_Na2S2O8_ is the Na_2_S_2_O_8_ cost, 5000 RMB/t.

According to Eq. (5), the operational cost at initial Na_2_S_2_O_8_ concentration at 1.5 mmol/L and 2.0 mmol/L was 160.5 RMB/kg and 214.9 RMB/kg, respectively. Compared with the degradation percentage of 95.20% and the energy consumption of 80.8 kWh/kg at initial Na_2_S_2_O_8_ concentration of 2.5 mmol/L, the degradation percentage of 90.42% at initial Na_2_S_2_O_8_ concentration of 1.5 mmol/L was a little lower with a little higher energy consumption of 85.1 kWh/kg. However, the initial Na_2_S_2_O_8_ concentration of 2.5 mmol/L exhibited Na_2_S_2_O_8_ cost of 156.3 RMB/kg that was much higher than 98.8 RMB/kg of the Na_2_S_2_O_8_ cost for that of 1.5 mmol/L. Considering the Na_2_S_2_O_8_ cost and the energy consumption cost, the initial Na_2_S_2_O_8_ concentration of 1.5 mmol/L was selected as one of the primary conditions in the following experiments.

### The effect of initial dye concentration on degradation performance

**Figure 8:**
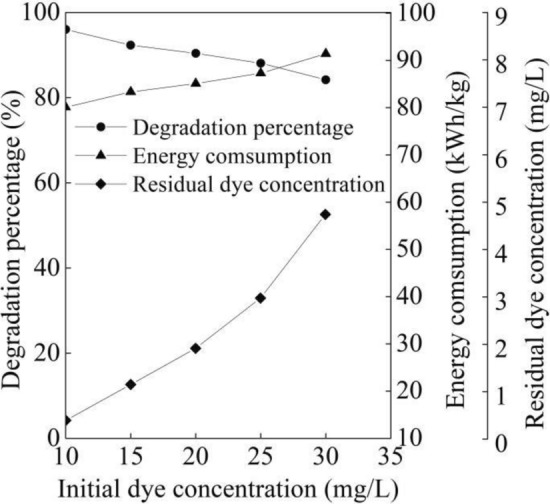
Degradation performance of different initial dye concentration.

As shown in Fig. 8, the degradation percentage of the B-3BF dye wastewater gradually decreased from 96.04 to 84.20% with initial dye concentration from 10 to 30 mg/L at six degradation times, flow rate of 500 L/h, initial pH of 7.8 and initial Na_2_S_2_O_8_ concentration of 1.5 mmol/L. In addition, it can be seen from Fig. 8 that the residual dye concentration after six degradation times increased with initial dye concentration from 0.40 to 4.74 mg/L from 10 to 30 mg/L. The residual dye concentration with the initial dye concentration of 20 mg/L reached the lowest value of 1.92 mg/L. The degradation percentage was linked to the increase of initial dye concentration due to the fixed amount of oxidant concentration at the same initial Na_2_S_2_O_8_ concentration and other experimental conditions. With the initial dye concentration from 10 to 30 mg/L, the residual dye that was not degraded under UV irradiation with Na_2_S_2_O_8_ increased under same experimental conditions. In addition, with the increase of initial dye concentration, the penetration of photons from the UV lamps entering into the solution was decreased, which lowered the generation of sulfate radicals and increased the residual dye. Consequently, the dye that could not be degraded was gradually increased with the increase of initial dye concentration, which resulted in the decrease of the degradation percentage. To our best knowledge, there lacks of the degradation of B-3BF dye by UV irradiation, which highlights research in the degradation of B-3BF dye by UV irradiation with persulfate in this work. With the initial B-3BF dye concentration of 50 mg/L and the contact time of 10 min, the degradation percentage of the B-3BF dye wastewater was reported to reach 92% by the oxidation combining modified pyrolusite and H_2_O_2_^34^. However, it requires further research in the performance stability of prepared modified pyrolusite before the modified pyrolusite is applied in the industrial degradation of textile wastewater. The energy consumption of the B-3BF dye wastewater gradually increased with initial dye concentration from 10 to 30 mg/L at six degradation times, flow rate of 500 L/h, initial pH of 7.8 and initial Na_2_S_2_O_8_ concentration of 1.5 mmol/L as shown in Fig. 8. The increase of residual dye concentration and the decrease of degradation percentage resulted in the increase of the energy consumption according to Eq. (2).

## Conclusions

Degradation performance of B-3BF dye wastewater by UV irradiation (185/254 nm) with sodium persulfate was more effective than those by UV irradiation (185/185 nm) and UV irradiation (254/254 nm) with sodium persulfate. The degradation percentage of B-3BF dye could reach 90.42% with the energy consumption of 85.1 kWh/kg and the residual dye concentration of 1.92 mg/L by low-pressure UV lamps at 185 nm and 254 nm with initial Na_2_S_2_O_8_ concentration of 1.5 mmol/L and initial dye concentration of 20 mg/L in the pilot UV device. The addition of Na_2_S_2_O_8_ concentration could dramatically improve the degradation performance of B-3BF dye wastewater. The experimental results suggested that the degradation of the B-3BF dye wastewater using UV irradiation (185/254 nm) with sodium persulfate would be effective in the industrial application.

## Data Availability

The original contributions presented in the study are included in the article and further inquiries can be directed to the corresponding author.
